# Chronic schistosome infection leads to modulation of granuloma formation and systemic immune suppression

**DOI:** 10.3389/fimmu.2013.00039

**Published:** 2013-02-20

**Authors:** Steven K. Lundy, Nicholas W. Lukacs

**Affiliations:** ^1^Graduate Training Program in Immunology, University of Michigan Medical SchoolAnn Arbor, MI, USA; ^2^Department of Internal Medicine-Rheumatology, University of Michigan Medical SchoolAnn Arbor, MI, USA; ^3^Department of Pathology, University of Michigan Medical SchoolAnn Arbor, MI, USA

**Keywords:** T helper lymphocytes, immune regulation, hygiene hypothesis, soluble egg antigen, sialyl Lewis^x^ glycans

## Abstract

Schistosome worms have been infecting humans for millennia, but it is only in the last half century that we have begun to understand the complexities of this inter-relationship. As our sophistication about the inner workings of every aspect of the immune system has increased, it has also become obvious that schistosome infections have broad ranging effects on nearly all of the innate and adaptive immune response mechanisms. Selective pressures on both the worms and their hosts, has no doubt led to co-evolution of protective mechanisms, particularly those that favor granuloma formation around schistosome eggs and immune suppression during chronic infection. The immune modulatory effects that chronic schistosome infection and egg deposition elicit have been intensely studied, not only because of their major implications to public health issues, but also due to the emerging evidence that schistosome infection may protect humans from severe allergies and autoimmunity. Mouse models of schistosome infection have been extremely valuable for studying immune modulation and regulation, and in the discovery of novel aspects of immunity. A progression of immune reactions occurs during granuloma formation ranging from innate inflammation, to activation of each branch of adaptive immune response, and culminating in systemic immune suppression and granuloma fibrosis. Although molecular factors from schistosome eggs have been identified as mediators of immune modulation and suppressive functions of T and B cells, much work is still needed to define the mechanisms of the immune alteration and determine whether therapies for asthma or autoimmunity could be developed from these pathways.

## Introduction

*Schistosoma mansoni, Schistosoma haematobium*, and *Schistosoma japonicum* are helminth worm species that infect humans and are highly prevalent in warm climates. They are obligate parasites that require a supply of blood from mammalian hosts to mature from larval stages to adult worms. Schistosomes live within the body of their hosts where they attach to the walls of intestinal blood vessels for feeding. Adult male and female schistosomes form copulating pairs that can produce as many as 300 eggs per couple per day. The eggs only hatch in fresh water, and the first larval form requires the presence of freshwater snails in order to mature into cercariae, the larval form that infects humans and other mammals. Unfortunately, a large portion of the eggs that are produced enter the portal circulation instead of leaving the body and deposit in internal organs, particularly the liver. As will be described in more detail later in this review, the immune system of the host recognizes schistosome eggs as foreign and responds with a local granulomatous response and systemic changes to immunity. The ability of adult schistosome worms to persist in their hosts and the resulting continual production of eggs and their antigens drives the adaptive immune system toward a highly regulatory response that has repercussions to overall immunity.

## Modeling of schistosome Egg granuloma formation in mice

Most of what is known about the mechanisms by which schistosome granulomas form and function has come from the use of mouse models, especially in response to *Schistosoma mansoni* eggs (Boros, 1989). Schistosome cercariae can infect most if not all mammalian hosts and mice have proven very useful for studying granulomatous responses due to significant similarities with the human immune system, the availability of a vast array of reagents, and the production of many immunogenetically altered mouse strains that aid in mechanistic studies. The classic model used to study granuloma formation involves infection with schistosome cercariae, the larval form that infects humans, either through skin exposure or direct subcutaneous injection into the mouse. Adult worm pairs produce eggs continuously resulting in asynchronous granuloma formation. The natural infection model has been very useful in the study of the dynamics of the immune response, pathology, and granuloma architecture, but has some limitations due to its asynchronous nature. To study temporal aspects of egg deposition and granuloma formation, other models were developed in which purified schistosome eggs, or egg antigens coated to beads or macromolecular compounds were injected into the tail veins of mice (Boros and Warren, 1971b). The intravenous injection model results in egg deposition primarily in the lungs, where the granulomas form simultaneously and temporal changes in the immune response can be more easily tracked. Differences have been noted between lung versus hepatic granulomas, and between the differing species of schistosome worms. Therefore, it is important to consider the route and form of administration, localization of granuloma formation, and the infectious agent when interpreting results.

## The schistosome Egg granuloma: a necessary evil

Schistosome eggs have an outer shell made of chitin that houses the larval form, miracidiae, which is responsible for the release of soluble egg antigens (SEA). The miracidiae do not hatch in the host tissues, but production of SEA while the larvae are still viable stimulates the host immune response to form a granuloma (Boros and Warren, 1970). Using the temporal induction models, it was determined that the initial response to egg deposition and SEA release in small blood vessels involves the local production of inflammatory cytokines (TNFα, IL-1) and chemokines from resident epithelial cells and macrophages (Joseph and Boros, 1993; Lukacs et al., 1993; Wynn et al., 1993; Burke et al., 2010). This triggers the early influx of monocytes, neutrophils, and lymphocytes and the establishment of schistosome egg granulomas. Each schistosome egg and its individual granuloma do not pose much of a threat to the host. Over time, however, the constant deposition of eggs and formation of granulomas leads to hepatosplenomegaly and significant blockage of portal blood flow (Boros, 1989). Portal hypertension promotes the development of intestinal and esophageal varices resulting in severe bleeding and eventually can lead to the death of the infected individual. Schistosome egg granulomas were therefore viewed as major contributors to the pathogenesis of schistosome infection and strategies were sought to inhibit or prevent granuloma formation.

Several lines of evidence demonstrated that granuloma formation in response to SEA was dependent on the activation of CD4^+^ T helper (T_H_) lymphocytes and also highlighted the importance of granuloma formation to host survival. Infection of athymic nude mice led to substantially decreased granuloma size and impairment of the anti-SEA antibody response (Phillips et al., 1977; Amsden et al., 1980). Elimination of T_H_ cells by treatment with anti-lymphocyte serum or the specific, complement-fixing anti-CD4 antibody, L3T4, led to decreased granuloma formation and IL-2 production by spleen cells of infected mice (Domingo and Warren, 1968; Mathew and Boros, 1986). The severe absence of granuloma formation following high efficiency anti-CD4-depletion in mice led to the influx of low numbers of macrophages or eosinophils, diminished collagen deposition around eggs and increased damage to local hepatocytes (Mathew and Boros, 1986; Fallon et al., 2000b). Further evidence of the critical importance of T_H_ cell-mediated granuloma formation came from studies of infection in thymectomized mice and mice receiving additional T cell ablative therapies in which severe inhibition of granuloma formation led to mortality due to increased liver damage (Lucas et al., 1980; Fallon and Dunne, 1999). Toxicity to hepatocytes in the absence of granuloma formation has been attributed to the hepatotoxic cationic glycoproteins, α_1_ and ω_1_, released by schistosome eggs as part of SEA (Dunne et al., 1991; Abdulla et al., 2011). The mechanisms by which granulomas prevent direct hepatotoxicity have yet to be shown definitively, but of potential interest is a recent finding that the glycosylated T2 ribonuclease ω1 binds to the mannose receptor, a C-type lectin expressed by macrophages and dendritic cells (DC) (Dewals et al., 2010; Everts et al., 2012). Binding and internalization of ω1 through the mannose receptor on DC leads to polarization of these antigen presenting cells toward a T_H_2 inducing phenotype (Everts et al., 2009, 2012). Therefore, infiltration of macrophages and DC into granulomas in the liver appears to be one host protective mechanism for binding and neutralizing hepatotoxic glycans from the schistosome egg and surrounding tissue and for the ultimate survival of the host. At the same time, the response to ω1 by DC and macrophages drives the T_H_2 immune response leading to increased lymphocyte and eosinophil infiltration and enlargement of the granuloma (Everts et al., 2009; Steinfelder et al., 2009). As will be discussed below, this T_H_2 dominated response may also be an adaptive mechanism of protection of the host from deleterious prolonged T_H_1 mediated immunity. Chronic schistosome infection leads to further modulation of the immune response going from an acute T_H_2 reaction toward immune regulation and fibrosis.

## Schistosome granuloma formation as a dynamic model of adaptive immune responses

### T_H_1 cells mediate early granuloma formation

**Figure 1:**
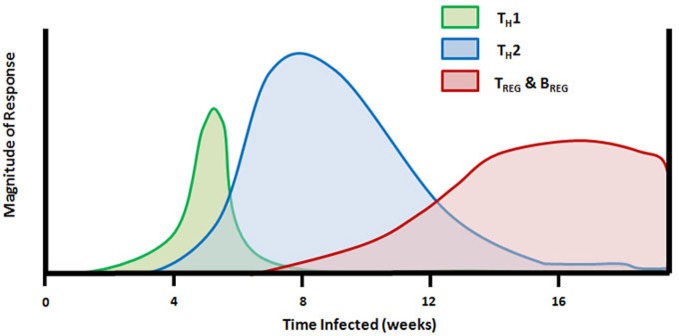
**Timeline of T_H_ cell mediated responses in the mouse model of schistosome infection.** Subcutaneous injection of schistosome cercariae on day 0 leads to development of adult worms and egg production beginning at 4–5 weeks. The early innate and adaptive immune response to adult worm antigens is dominated by proinflammatory and T_H_1 cytokines (TNFα, IL-12, and IFNγ). Following egg deposition in the liver and other internal organs, the larval miracidiae release soluble egg antigens (SEA) containing molecules that drive a rapid transition from T_H_1 to T_H_2-dominated immunity and production of IL-4, IL-5, and IL-13. Between 7 and 8 weeks of infection, FoxP3^+^ T_REG_ cells and IL-10 are detectable, and the population of splenic FasL^+^ CD5^+^ B cells begins to proliferate. T_H_2 response and peak granuloma formation occurs between 8 and 10 weeks of infection and is followed by granuloma downmodulation and increasing fibrosis around newly deposited eggs which persists throughout the remainder of infection.

The role of T_H_ cell-derived cytokines in schistosome granuloma formation has been intensively studied and reviewed (Scott et al., 1989; Boros, 1994; Milner et al., 2010). A timeline of the changes in mouse T_H_ cell responses during the natural infection by schistosome cercariae is given in Figure 1. Following the initial proinflammatory cytokine release, CD4^+^ T_H_ cells enter the lesion and release T_H_1-type cytokines, IL-2 and IFNγ, which facilitates the establishment of delayed-type hypersensitivity response and early granuloma formation. A series of experiments led to the discovery of antigenic peptides, some that were able to induce T_H_1-type immune responses even in the absence of an adjuvant (Lukacs and Boros, 1991, 1992, 1993; Cai et al., 1996; Chen and Boros, 1998, 2001; Reis et al., 2008). The finding that neutralization of IL-4 during schistosome infection led to decreased granuloma formation prompted interest in prolonging T_H_1 responses during schistosome infection in order to reduce pathology (Yamashita and Boros, 1992). Initial results indicated that IL-12 could drive expression of IFNγ, IL-2, IL-10, and IL-12 in pulmonary granulomas and could reverse established T_H_2-type responses in egg pre-sensitized animals (Wynn et al., 1994). Further studies indicated that vaccination of mice with eggs plus IL-12 led to decreased fibrosis similar to that seen in Stat-6 knockout mice that lacked IL-4 receptor signaling (Wynn et al., 1995; Kaplan et al., 1998). Much of the IL-12 adjuvant effect was attributed to induction of IFNγ (Boros and Whitfield, 1998). However, extreme skewing of the immune response toward schistosome eggs to a T_H_1-type reaction resulted in increased mortality and liver pathology (Boros and Whitfield, 1998; Fallon and Dunne, 1999; Wynn and Hoffmann, 2000). In addition to the deleterious effects of an overt T_H_1-type response in mice, it has also been shown that the more severe form of human disease, hepatosplenic schistosomiasis, is associated with elevated levels of TNFα and IFNγ and lower levels of the T_H_2-type cytokine IL-5 (Mwatha et al., 1998). Thus, although decreased granuloma size and fibrosis in T_H_1 dominated responses appeared to be desirable, it is clear that the switch to a T_H_2-type response may have some protective effect for the host.

### T_H_2 cells and non-T cell sources of IL-4

In the murine model, the cytokine response to worm eggs and SEA begins to switch to a T_H_2-type response during the seventh week post-infection. By 8 weeks of infection, the production of IL-4 and other T_H_2 cytokines predominates and IFNγ is barely detectable. The switch to a T_H_2-type reaction is accompanied by a change in the cellularity of the granuloma with a dramatic influx of eosinophils, mast cells, and fibroblasts into the lesion. The influx of these cell types results in enlarged granulomas with increasing fibrosis. The mechanisms underlying this switch in cytokine pattern have been intensely studied. Neutralization of IL-4 or infection of IL-4-deficient or IL-4R-deficient mice leads to decreased T_H_2-type cytokine production and prolonged T_H_1 responses (Cheever et al., 1994; Kaplan et al., 1998; Jankovic et al., 1999). However, in certain murine strains, IL-4 deficiency was not enough to drive substantial increases in IFNγ production or to completely prevent development of a T_H_2-type response during infection (Metwali et al., 1996; Rakasz et al., 1998). IL-10 and/or TGF-β were the dominant mediators T_H_1 downregulation in IL-4 deficient mice (Rakasz et al., 1998). IL-4 deficient mice display reduced expression of IL-13, but the residual IL-13 may play a compensatory role for the loss of IL-4 expression (Chiaramonte et al., 1999b; McKenzie et al., 1999). IL-13 plays a similar role as IL-4 through binding to the IL-4 receptor and participates in granuloma formation, IgE induction, and inhibition of IFNγ production. In contrast to IL-4, IL-13 is also involved in the induction of collagen synthesis by granuloma fibroblasts through binding to the specific IL-13R (Chiaramonte et al., 1999a; Fallon et al., 2000a). T_H_1 and T_H_2 cytokines participate in cross-regulation of synthesis and function of the opposing cytokine response, therefore, it was initially unclear how the T_H_2 response could be induced while the T_H_1-type response that downregulates it was already actively mediating granuloma formation. One possibility is that IL-12 production from granuloma macrophages is decreased upon exposure to SEA (Boros, 1999; Todt et al., 2000).

Lacto-N-fucopentaose III (LNFPIII), a glycan component of SEA, has been shown to act directly on DC and monocytes to favor the development of the T_H_2 response (Wang et al., 2010; Zhu et al., 2012). In comparison to LPS stimulation, LNFPIII induced an increase in the ratio of CD86/CD80 expression on DC, a similar pattern of chemokine expression, higher expression of IL-6 but no IL-12 from purified DC (Wang et al., 2010). Co-culture of these LNFPIII-stimulated DC with naive OT-II T_H_ cells led to dominant production of IL-4 and no IFNγ, but induced similar levels of IFNγ and cytolytic activity of OT-I CD8^+^ T_C_ cells following co-culture in comparison with LPS-stimulated DC (Wang et al., 2010). LNFPIII was also shown to program macrophages to induce IFNγ production from NK cells by an IL-12-independent, but CD40/CD40L-dependent mechanism. Treatment with dextran conjugated to LNFPIII was successful at inhibiting experimental autoimmune encephalomyelitis (EAE) in mice through a mechanism involving alternative activation of CD11b^+^Ly-6C^high^ monocytes (Zhu et al., 2012). The LNFPIII-treated TCR transgenic 2D2 mice had elevated levels of IFNγ as well as IL-4, IL-5, IL-13, and IL-10 in response to immunization with MOG peptide in comparison with mice that received unconjugated protein (Zhu et al., 2012). These data demonstrate alternate pathways by which LNFPIII elicits both IFNγ production from NK and CTL cells while also mediating a T_H_2 and T_REG_ dominated responses from CD4^+^ T cells, and suggest a mechanism for the induction of T_H_2 cells in the face of a strong T_H_1 immune response. The T2 ribonuclease, ω1, component of SEA was demonstrated to directly inhibit the activation of DC by LPS, suggesting that it is a critical factor in the inhibition of the T_H_1 response (Everts et al., 2009; Steinfelder et al., 2009).

A study done using SEA stimulation of human basophils was one of the first in which IL-4 production by these cells was definitively shown (Falcone et al., 1996). Since that time it has become evident that basophils are not only capable of producing IL-4 but are also important antigen presenting cells that can initiate T_H_2 responses (Perrigoue et al., 2009; Sokol et al., 2009; Yoshimoto et al., 2009). The IPSE/α-1 glycoprotein component of SEA was shown to activate IL-4 production from mouse basophils through a mechanism dependent on IgE antibodies and FcΣR expression (Schramm et al., 2007). Several other groups have also demonstrated IL-4 production in response to SEA by non-CD4^+^ peritoneal exudate cells and activated granuloma eosinophils (Williams et al., 1993; Sabin and Pearce, 1995; Kullberg et al., 1996; Sabin et al., 1996b; Rumbley et al., 1999). IL-5 is a stimulatory cytokine for eosinophils and studies in IL-5 deficient mice have revealed a reduction in IL-4 production from ionomycin stimulated non-B, non-T cells from infected mice (Brunet et al., 1999). However, IL-4 production from SEA-stimulated or IgE crosslinked non-B, non-T cells of IL-5 deficient mice was comparable to normal mice and anti-IL-5 antibody treatment did not reduce granuloma sizes in infected mice (Sher et al., 1990). These non-T cell sources of IL-4 may be sufficient to supply the early IL-4 necessary to down regulate IL-12 and IFNγ production and to stimulate T_H_2 cell differentiation.

### The role of T_H_17 cells in granuloma pathology

The first evidence of a role for IL-17 in schistosome granuloma formation came from a study in which blockade of the IL-12p40 receptor subunit shared by IL-12 and IL-23, but not the IL-12p35 receptor subunit that is specific to IL-12, led to decreased granuloma size (Rutitzky et al., 2005). Granuloma formation correlated with levels of IL-17 production in susceptible vs. resistant strains of mice and treatment of susceptible strains with anti-IL-17 neutralizing antibodies led to decreases in granuloma size (Rutitzky et al., 2005). It was later determined that SEA had direct stimulatory effects on IL-23 and IL-1 production by dendritic cells (Rutitzky et al., 2008; Shainheit et al., 2008). Mice with genetic deficiencies of the T_H_1-specific transcription factor T-bet or IFNγ displayed enlarged schistosome granulomas and elevated IL-17 levels (Rutitzky et al., 2009; Rutitzky and Stadecker, 2011). The studies described above were mostly performed using immunization with SEA and complete Freund's adjuvant in the normally granuloma resistant C57BL/6 strain of mice. IL-4 and type 1 interferons, which may be suppressed by instillation of complete Freund's adjuvant, are normally elicited by schistosome eggs and are known inhibitors of T_H_17 cells. It will be interesting to determine whether IL-4 and IFNα or IFNβ display inhibitory effects on IL-23 or IL-17 during schistosome infection in granuloma susceptible mice.

### B lymphocytes and antibodies

The dynamics of total B cell distribution and their roles during schistosome infection have also been intensely investigated. It was found that the absolute numbers of B cells in the spleen, lymph nodes and blood increased dramatically during the acute T_H_2 phase of infection and remained high throughout the chronic infection (Chensue and Boros, 1979; El-Cheikh et al., 1998). The absolute number of T cells rose slightly in the same organs leading to a relative decrease in the number of T cells compared to B cells. This expansion of B cells was polyclonal in nature yielding both antigen-specific and non-specific antibodies, including autoreactive antibodies (Fischer et al., 1981; Lopes et al., 1990). SEA-specific immunoglobulin production begins following egg deposition and increases throughout the acute and chronic downmodulated phases of schistosome infection (Boros et al., 1975). Evidence of intra-granulomatous antibody production exists with IgG1 being the predominant antibody isotypes released from acute-phase granulomas and a mixture of IgG1, IgG2a, IgG2b, IgG3, and IgA released by chronic-phase granulomas (Boros et al., 1982). Intralesional production of IgM peaked at 12–16 weeks of infection while IgG production was highest at 20 weeks of infection (Boros et al., 1982). The role of antibodies in the pathogenesis of schistosome granuloma formation is complex, with several studies showing an active immune regulatory role of antibodies from infected individuals (Goes et al., 1991; Jankovic et al., 1997, 1998; Rezende et al., 1998). Immune complexes from the blood of chronically infected human patients were able to inhibit *in vitro* granuloma formation (Goes et al., 1991). This inhibition was dependent on the presence of the Fc portion of the immunoglobulin and could be reversed by treatment with indomethacin, indicating a role for prostaglandins in immune suppression mediated by immune complexes (Goes et al., 1991). Infection of mice deficient in either FcγR or FcΣR led to increased size and collagen content of acute-phase and chronic granulomas (Jankovic et al., 1997, 1998). SEA-specific antibodies may mediate granuloma downmodulation by arming FcR positive suppressor cells, or by neutralizing and sequestering antigens. Another mechanism by which immune complexes may mediate downmodulation is through inhibition of MHC Class II expression and disruption of antigen presentation (Rezende et al., 1998).

Several lines of evidence suggest that immune regulation and control of granuloma formation may be the primary function of B cells during schistosome infection. One study demonstrated that SEA-stimulated mesenteric lymph node cells of B cell-deficient (JHD) mice produced higher amounts of IFNγ and IL-12 cytokines and reduced amounts of IL-4 and IL-10 when stimulated with SEA (Hernandez et al., 1997). Irradiated splenocytes from JHD mice were more effective at eliciting a T_H_1-type response from SEA-specific CD4^+^ T cells. Another study in B cell-deficient (μMT) mice showed increased mRNA expression of T_H_1 cytokines in B-deficient compared to wild-type mice (Ferru et al., 1998). These authors concluded that the B cell population was necessary to drive T_H_2-type responses during infection. However, a different study using μMT mice demonstrated only a reduction in IL-4 production without differences in IL-5, IL-10, or IFNγ production (Jankovic et al., 1998). In contrast, infection of BALB.Xid mice, which have a deficiency in B cell receptor signaling and severe reductions in the mucosal CD5^+^ B cell compartment, revealed a defect in antigen-stimulated IL-10 production, reduced SEA-specific IgM and IgA titers, increased IFNγ and IL-4 production, elevated IgE and IgG1 titers, increased tissue egg burdens and higher mortality (Gaubert et al., 1999). Thus, B cells appear to play a central role in mediating the transition from T_H_1 to T_H_2 responses and in the regulation of the granulomatous response during acute and chronic infection. Antibody-independent regulatory mechanisms of B cells are discussed below.

## Systemic immune regulation stimulated by chronic schistosome infection

As the T_H_2 response progresses, SEA-induced and cytokine-mediated collagen synthesis by granuloma fibroblasts leads to increased hepatic fibrosis that continues through the remainder of infection (Boros and Lande, 1983). Schistosome granulomas reach peak size and cellularity at 8–10 weeks of natural murine infection. After 10 weeks, a spontaneous diminution of cytokine responses to SEA and decreased granuloma size (granuloma downmodulation) is observed leading to a less severe chronic stage of the murine disease by 14 weeks (Boros et al., 1975; Colley, 1975). Downmodulation is accompanied by cumulative hepatic fibrosis and elevated anti-SEA antibody titers (Boros et al., 1975). Splenectomy of infected mice at 8 weeks of infection led to increased granuloma size at 12 weeks of infection indicating that a splenic mechanism was involved in granuloma downmodulation (Hood and Boros, 1980). Subsequent studies were directed at identifying the splenic factor(s) involved in granuloma downmodulation.

The earliest findings showed that spleen cells from chronically infected mice were able to transfer downmodulation to acutely infected mice (Colley, 1976; Chensue and Boros, 1979). Depletion of T cells from spleen cell preparations demonstrated their role in transferring downmodulation (Chensue and Boros, 1979). A complex series of experiments followed in which the granuloma inducing properties of T cells were attributed to T_H_ cells, while granuloma and SEA-induced suppressive effects were attributed to both the T_H_ and CD8^+^ cytotoxic T lymphocyte (CTL) subsets (Chensue et al., 1981; Weinstock and Boros, 1983; Ragheb and Boros, 1989; Fidel and Boros, 1990). However, a subsequent study of infection in CTL-deficient mice demonstrated normal granuloma formation during acute infection and normal downmodulation during chronic infection (Yap et al., 1997). These data suggested that although highly purified CTL were capable of regulating granuloma formation, their presence *in vivo* was not critical for controlling the immune response toward schistosome eggs. This indicated the importance of CTL-independent factors in mediating granuloma downmodulation that are now understood to involve the activation of regulatory populations of T and B lymphocytes as well as other mechanisms of immune suppression.

### Regulatory cytokines: IL-10 and TGF-β

The anti-inflammatory cytokine product of regulatory lymphocytes, IL-10, was recognized as an important mediator of the switch from T_H_1- to T_H_2-type response during schistosomiasis in the early 1990s (Sher et al., 1991; Oswald et al., 1992b). As is now a widely recognized property of IL-10, early neutralization studies led to increased MHC Class II and B7-1/B7-2 costimulatory molecule expression on granuloma macrophages and increased antigen presentation capacity for T_H_1 cells (Flores Villanueva et al., 1993). In a subsequent study, recombinant IL-10 or IL-10/Fc fusion protein treatment of infected or egg-injected mice led to decreased granuloma formation, decreased IL-2 and IFN-γ production, and increased IL-4 and IL-10 production (Flores-Villanueva et al., 1996). In one study, mice treated with anti-IL-10 antibodies *in vivo* displayed increased hepatic and pulmonary granuloma size, increased eosinophilia, and elevated IFN-γ and IL-5 levels during acute infection (Boros and Whitfield, 1998). However, another study indicated that granuloma downmodulation was not impaired in IL-10 deficient mice (Wynn et al., 1998). The same group also showed that infections of IL-10/IL-4 and IL-10/IL-12 double deficient mice in comparison to mice with singular deficiencies led to severe immune polarizations to T_H_1 and T_H_2, respectively (Wynn et al., 1997). The double deficient mice died from very distinct pathologic mechanisms with the IL-10/IL-12 mice that were heavily skewed toward the T_H_2 response having enlarged and highly fibrotic granulomas (Wynn et al., 1997; Hoffmann et al., 2000). These data indicated that IL-10 regulates the T_H_2 as well as the T_H_1 response toward schistosome antigens. Production of IL-10 in response to stimulation with SEA or its major glycan component, lacto-N-fucopentaose III (LNFPIII), has been attributed to T_H_ cells, B cells and monocytes (Sher et al., 1991; Velupillai et al., 1996a, 1997; Terrazas et al., 2001).

The role of TGFβ in granuloma pathology is even more complex than that of IL-10. TGFβ has an inhibitory effect on macrophages and T_H_1 cells in the schistosome model (Oswald et al., 1992a; Qadir et al., 2001). Studies of inhibition of T_H_ cell responses and schistosome granuloma formation by TGFβ have the limitation that IL-10 and IL-4 were usually present (Oswald et al., 1992a; Qadir et al., 2001). In contrast, TGFβ in the context of proinflammatory cytokines acts as an important cofactor for T_H_17 cell differentiation, and therefore, can contribute to the enhanced granuloma formation described in a previous section (Shainheit et al., 2008). TGFβ also induces the production of connective tissue components by fibroblasts and therefore may play a role in the development of fibrosis and portal hypertension seen in severely infected individuals (Wahl et al., 1997).

### Regulatory T lymphocytes

With the discovery that FoxP3 was the canonical transcription factor for regulatory T (T_REG_) cells that produced IL-10 and TGFβ, it was of interest to study whether the previously observed effects of chronic granuloma-induced immune suppression were attributable to T_REG_ cells (Hori et al., 2003). In the natural infection model with *S. mansoni*, dramatic and progressive increases in the expression of FoxP3 mRNA were observed in the liver and spleen, with peak expression occurring at the 16 week granuloma downmodulated stage and highest in the liver (Singh et al., 2005; Taylor et al., 2006). Additional phenotypic markers of T_REG_ cells (CD103, GITR, OX40, and CTLA-4) were also elevated at the mRNA level in the spleens of schistosome-infected mice (Layland et al., 2010). Over-expression of FoxP3 through retroviral gene transfer at the beginning of egg deposition resulted in increased expression of IL-4, IFNγ, and TGFβ but not IL-10, yet resulted in decreased granuloma formation (Singh et al., 2005). The expansion but low IL-10 expression of FoxP3^+^ T_REG_ cells was also observed in a separate study of the natural infection model (Baumgart et al., 2006). Transfer of immune tolerance by T_REG_ derived from schistosome infected hosts was IL-10 independent (Pacifico et al., 2009). These data demonstrate that T_REG_ cells are activated and recruited by the schistosome granuloma, but that they are not necessarily the main source of IL-10 during infection, nor are they dependent on IL-10 for their immune suppressive functions.

### Regulatory B lymphocytes

The realization that B cells have regulatory capacity was initially demonstrated in response to sheep red blood cells and was associated first with regulatory antibody production and later linked to interactions with T cells (Stockinger et al., 1979; Zubler et al., 1980). Subsequent studies in mice and humans have demonstrated that B lymphocytes can regulate immune responses through antibody-dependent mechanisms as well as via direct cell–cell interactions and regulatory cytokine production (Klinker and Lundy, 2012). The schistosome granuloma model was one of the first natural infection models in which the regulatory properties of B lymphocytes were reported (Cheever et al., 1985). Mice that were genetically deficient in B cells failed to downmodulate granuloma formation around eggs deposited in the liver during chronic infection and had higher mortality rates than control mice with normal B cell development (Cheever et al., 1985). The regulatory properties of B cells in schistosome infection were originally attributed to the increased production of natural antibodies, which more recently have been demonstrated to play a role in the clearance of apoptotic cell debris and immune suppression (Chen et al., 2009; Vas et al., 2012). In addition, it was demonstrated that IL-10 production by B cells, particularly the CD5^+^ B-1a cell subset, was induced *in vitro* by the schistosome glycan LNFPIII found in SEA (Velupillai and Harn, 1994; Velupillai et al., 1996a, 1997). A study of schistosome infection in Xid mice, which have a severe deficiency in CD5^+^ B cells, demonstrated that this subset plays a role in regulating the production of IFNγ and IL-5 by T_H_ cells and in controlling granuloma size (Gaubert et al., 1999). IL-10 acts as an autocrine growth factor for CD5^+^ B cells and both IL-12 and IFNγ inhibit the expansion of CD5^+^ B cells during schistosome infection (Velupillai et al., 1996b). Redistribution of splenic CD5^+^ B cells to Peyer's patches and mesenteric ganglia during schistosome infection has been reported, and may be one mechanism by which helminth infections regulate inflammatory bowel disease (El-Cheikh et al., 1998; Elliott et al., 2000).

Although most subsets of APC can produce IL-10 in response to stimulation through CD40 and toll-like receptors, two subsets of B cells have been identified as the major producers of IL-10 in mice, B10 and T2-MZP B cells (Evans et al., 2007; Yanaba et al., 2008). B10 cells have the surface phenotype CD5^+^CD1d^high^ and may be the cells that responded to stimulation with schistosome LNFPIII in the previous study (Velupillai et al., 1997; Yanaba et al., 2008). Stimulation of IL-10 production by T2-MZP cells, which have the phenotype CD21^high^CD23^+^, has not yet been studied during schistosome infection. B10 and T2-MZP cells have been used to transfer immune suppression in several models of inflammation in mice, and work is being done to identify the equivalent of IL-10 producing B10 and T2-MZP cells in human peripheral blood (Blair et al., 2010; Iwata et al., 2011). Importantly, the regulatory responses induced by B10 and T2-MZP cells appear to be antigen specific, despite being linked primarily to IL-10 production, suggesting the requirement for antigen presentation in their regulatory function. A recent study demonstrated IL-10 producing CD1d^high^ B cells from the spleens of schistosome infected mice could transfer immune regulation in a mouse model of ovalbumin-induced asthma (Van Der Vlugt et al., 2012). A similar population of B cells was found to be prevalent in the peripheral blood of children infected with *S. haematobium* in Gabon (Van Der Vlugt et al., 2012). The effect of schistosome infection on responses to other infections and inflammatory stimuli will be discussed in greater detail in another section.

### Activation-induced cell death and FasL^+^ B lymphocytes

Interestingly, the CD5^+^ B cells in schistosome-infected mice also expressed Fas ligand and were able to kill SEA-stimulated T_H_ cells by activation-induced cell death (AICD) (Lundy and Boros, 2002). AICD is a form of programmed cell death (apoptosis) that is an important mechanism of immune regulation during and after inflammatory reactions. Clusters of apoptotic cells were found in the spleens and granulomas of schistosome infected mice to a much greater extent than in spleens of uninfected mice (Estaquier et al., 1997). Culture of cells extracted from schistosome-infected mice demonstrated that CD4^+^ and CD8^+^ T cells spontaneously underwent apoptosis *in vitro* and were sensitive to apoptosis induction in response to IL-10 and mitogenic stimulation (Estaquier et al., 1997). A different study demonstrated increases in *in vivo* CD4^+^ and CD8^+^ T cell apoptosis from 4 to 7 weeks of infection compared to later time points, supporting the hypothesis that T_H_1 cells were more susceptible to AICD than their T_H_2 counterparts during schistosome infection (Fallon et al., 1998). In contrast, a study of human schistosomiasis indicated that T cells from patients with the less severe, intestinal form of disease were susceptible to apoptosis while T cells from patients with the more severe, hepatosplenic T_H_1 form of disease were resistant to egg antigen-induced apoptosis (Carneiro-Santos et al., 2000). In a more prolonged model of natural murine *Schistosoma mansoni* infection, systemic sensitivity to T_H_ cell apoptosis commenced soon after egg deposition (5 weeks), and progressed throughout the early granulomatous stages (6–10 weeks), in parallel with increased inflammation, and persisted at lower levels throughout the chronic, downmodulated stage (12–16 weeks) of the infection (Lundy et al., 2001). Levels of apoptosis exceeded 20% of the total CD4^+^ T cell population, which was far in excess of the expected number of antigen-specific cells, suggesting that a large number of bystander CD4^+^ T cells may be eliminated during schistosome infection (Lundy et al., 2001). Culture of splenocytes from infected and uninfected mice with SEA led to T_H_ cell apoptosis only in cells isolated from infected mice (Lundy et al., 2001). The three major lymphocyte populations of the spleen, CD4^+^ and CD8^+^ T cells as well as CD19^+^ B cells expressed the death-inducing molecule Fas ligand (FasL, CD178) (Lundy et al., 2001). The staining of freshly isolated splenocytes from 5 to 16 weeks infected mice demonstrated that the number of surface FasL^+^ cells correlated with the level of T_H_ cell apoptosis at each time point (Lundy et al., 2001).

FasL expression on CD8^+^ and CD4^+^ T cells is not surprising given that both T cell recognition of schistosome antigens and FasL up-regulation are mediated through the TCR. FasL expression by B cells was a much rarer finding, and the schistosome model provided some of the first evidence of the ability of isolated B lymphocytes to kill T_H_ cells in an SEA-dependent fashion (Lundy et al., 2001; Lundy and Boros, 2002; Lundy, 2009). Culture of splenocytes from 8 week-infected mice with SEA led to upregulated FasL expression on B and T cells and increased T_H_ cell apoptosis. Depletion studies indicated that the majority of T_H_ cell death was mediated by B cells rather than CD8^+^ CTL (Lundy et al., 2001). In a follow up study, B cell FasL expression was observed primarily on the splenic CD5^+^ B cell subset throughout schistosome infection and was even observed on splenic CD5^+^ B cells in uninfected mice (Lundy and Boros, 2002). Purified CD5^+^ B cells killed T_H_ cells from schistosome-infected mice at a low effector:target (0.5:1) ratio only when SEA was present (Lundy and Boros, 2002). FasL expression on CD5^+^ B cells was stimulated by culture with SEA and further enhanced when IL-4 and/or IL-10 were added to the culture (Lundy and Boros, 2002). The induction of IL-10 production by CD5^+^ B cells following exposure to schistosome glycans may act in an autocrine manner to induce higher FasL expression on these cells and suggests that CD5^+^ B cells may have several mechanisms of immune suppression at their disposal (Velupillai et al., 1996a; Klinker and Lundy, 2012). Induced FasL expression and IL-10 production by CD5^+^ B cells, which are well-documented to have autoreactive and poly-reactive antibody specificities, may be an important mechanism behind the correlations between schistosome infection and protection from autoimmunity and asthma described below (Lundy et al., 2005; Lundy and Fox, 2009).

### Other mechanisms of immune regulation

Other cell types may also participate in downregulation of the immune response to schistosome eggs. Granuloma macrophages represent about 30 percent of the total cells in the lesion. Early in the response to infection, macrophages and monocytes are important in the elicitation of delayed-type hypersensitivity granuloma formation and have cytotoxic effects on the miracidiae (Boros and Warren, 1971a). However, as described above, exposure to components of SEA results in alternative activation of monocytes and macrophages as well as DC, and promotion of the transition to a T_H_2 dominated immune response (Zhu et al., 2012). In addition, exposure of monocytes/macrophages to SEA leads to suppressive effects on SEA-induced T lymphocyte proliferation (Elliott and Boros, 1984). These macrophage suppressive effects may be attributable to SEA-induced production of IL-10, type 1 interferons and/or prostaglandin E_2_ (Elliott et al., 1987, 1990; Atochina and Harn, 2005). An interesting study of the effects of schistosome infection on chemically-induced experimental colitis in mice demonstrated an immune regulatory effect of F4/80^+^ macrophages that had migrated to the lamina propria (Smith et al., 2007). These macrophages were not induced by injection of schistosome eggs alone, and their mechanism of action was reported to be independent of alternative activation markers, interactions with T_H_2 and T_REG_ cells, or production of TGFβ or IL-10 (Smith et al., 2007). Schistosome infection also induces production of IL-10 by DC that in turn can induce the activation of FoxP3^+^ T_REG_ cells (Liu et al., 2011). These schistosome-induced DC transferred resistance to airway inflammation following adoptive transfer in an ovalbumin-induced model of asthma (Liu et al., 2011). Similar IL-10 production by DC and DC-dependent induction of T_REG_ cells was found following schistosome infection in the NOD mouse model of type 1 diabetes (Zaccone et al., 2009).

## Schistosome infection modulates immune responses toward other antigens

The systemic immune modulatory and regulatory pathways elicited by schistosome infection are not limited to antigens produced by adult schistosomes or their eggs (Harnett and Harnett, 2006; Helmby and Bickle, 2006; Kamal and El Sayed Khalifa, 2006; Zaccone et al., 2006). Bystander immune modulation by helminths has become a topic of great interest because of its negative impact on responses to vaccinations and pathogenic co-infections (Kamal and El Sayed Khalifa, 2006). With over 200 million estimated cases of schistosome infection worldwide, diminished responsiveness to protective vaccines and pathogenic infections poses a major public health hurdle, and justifies the support for global initiatives aimed at reducing schistosome infection (Borkow and Bentwich, 2000). Yet, as will be discussed, schistosomes and other helminths have been correlated to protection from allergies and autoimmunity by many epidemiological studies as well as experimental model systems and clinical trials (Kamal and El Sayed Khalifa, 2006; Zaccone et al., 2006; Maizels, 2009).

### Responses to vaccinations and concurrent infections

The switch from T_H_1- to a strong T_H_2-dominated immune response that occurs during progression of schistosome infection and egg granuloma formation has a negative impact on the ability of the host to respond correctly to intracellular pathogens. As the infection becomes more chronic, the T_H_2 response gives way to a highly regulatory immune response that can dampen both T_H_1 and T_H_2 immunity, leaving the host very susceptible to both intracellular and extracellular microbes. While this progression may be necessary to regulate liver toxicity and granuloma formation caused by the schistosome eggs (see above), it also causes significant morbidity and increased mortality in infected individuals. Schistosome infection has been shown to have preventive effects on immune responses elicited by vaccines against tetanus toxoid in humans and diphtheria toxin as well as Bacillus-Calmette-Guérin in mice (Sabin et al., 1996a; Haseeb and Craig, 1997; Elias et al., 2005). These examples illustrate altered humoral and cellular immune responses toward vaccines and will most likely translate to many other types of vaccinations including those against HIV, hepatitis viruses, *Mycobacteriae spp*. and malaria, significant pathogens that coexist in schistosome endemic areas.

The interaction between schistosome and HIV infections is of particular importance as the overlapping endemic regions for these two infections persist. Cells from schistosome infected individuals are more susceptible to HIV-1 infection reportedly due to IL-4 or IL-10 induced expression of the HIV co-receptors CXCR4 and CCR5 on immune cells of schistosome infected subjects (Shapira-Nahor et al., 1998; Kalinkovich et al., 1999; Secor et al., 2003). Selective inhibition of the cytolytic response of CD8^+^ T cells for the HIV Gag protein has also been noted in schistosome infected individuals (McElroy et al., 2005). These findings could be expected to increase cell–cell transmission and viremia in individual patients, and therefore, to increase the person to person transmission of HIV in schistosome endemic areas (Quinn et al., 2000). However, studies of HIV viral load and disease progression in schistosome infected individuals following de-worming have yielded conflicting results (Lawn et al., 2000; Wolday et al., 2002; Secor et al., 2004; Kallestrup et al., 2005). Susceptibility to HIV infection and response of the virus to de-worming is undoubtedly dependent on the stage and severity of the schistosome infection and the status of the immune response at the time of HIV exposure. In contrast, schistosome egg excretion is increased by T cell-mediated inflammation, and persons co-infected with HIV had marked decreases in the number of excreted eggs (Doenhoff, 1997).

Another major public health concern is co-infection of schistosome infected individuals with liver tropic viruses such hepatitis B and C (Kamal et al., 2004). Although there is evidence that schistosome infection does not increase the incidence of hepatitis infection in endemic areas for both diseases, co-infections in these regions are quite frequent (Larouze et al., 1987; Pereira et al., 1994; Angelico et al., 1997). Interestingly, a study of liver biopsies of patients infected with hepatitis C showed no significant impact of co-infection by schistosomes or the presence of egg granulomas on virus-induced pathology (Helal et al., 1998). However, a separate study focused instead on the liver pathology of schistosome infected individuals, showed that co-infection with hepatitis C led to much more severe liver damage including cirrhosis and cancers that were not found in the absence of HCV co-infection (Mohamed et al., 1998). Despite the lack of evidence that schistosome co-infection changes the course of liver pathology caused by hepatitis virus, some studies have shown differences in T_H_ cell cytokine responses toward viral antigens in schistosome co-infected people and viral persistence (Kamal et al., 2001; El-Kady et al., 2005). Schistosome co-infection suppressed the T_H_1 response normally elicited by HCV infection (Kamal et al., 2001; El-Kady et al., 2005). An improvement was seen in the anti-viral response of a cohort of co-infected patients following treatment with praziquantel to remove schistosomes (Farghaly and Barakat, 1993). In an experimental model of hepatic virus infection, schistosome infected mice co-infected with LCMV had an influx of activated CD8^+^ CTL cells expressing IFNγ into the liver, which led to down regulation of the granuloma T_H_2 response (Edwards et al., 2005). The end result of LCMV co-infection was increased liver toxicity and viral replication in the schistosome infected mice (Edwards et al., 2005). Thus, schistosomes and hepatotropic viruses have a complex relationship that generally leads to alterations in immune responses to both pathogens, and worse prognosis.

Schistosome infections can also influence the immune response toward other pathogens. Mice co-infected with *Mycobacterium avium* and *Schistosoma mansoni* had a shift to T_H_2 dominated cytokine responses toward mycobacterial antigens even when the *M. avium* infection was established first (Sacco et al., 2002). Similar results were seen in a co-infection model of *S. mansoni* and *M. leprae* in mice (Frantz et al., 2010). As noted above, schistosome infected mice responded poorly to vaccination with Bacillus-Calmette-Guerin, yet in the *M. leprae* study, vaccination with a DNA vaccine encoding the heat shock protein 65 molecule did induce a protective response in *S. mansoni* infected mice (Elias et al., 2005; Frantz et al., 2010). It remains to be seen whether such a strategy could be effective in co-infected humans, particularly those individuals who are also infected with the HIV virus. A study of the progression of active tuberculosis in HIV-infected individuals revealed a correlation between co-infection with schistosomes and increased active tuberculosis (Brown et al., 2006). A notable increase in susceptibility to malaria infection has also been described in people infected with schistosomes and other intestinal helminths (Nacher et al., 2002b; Sokhna et al., 2004). At the same time, schistosome infection may play a beneficial role in limiting the more severe cerebral form of malaria infection (Nacher et al., 2002a; Waknine-Grinberg et al., 2010). Co-infection of mice with *Plasmodium chabaudi* and *S. mansoni* led to reciprocal alterations in the immune response to either pathogen (Helmby et al., 1998). The widespread presence of schistosomes in areas that are endemic for these other major pathogens is likely to impede progress in designing effective vaccination and eradication strategies in the near future.

### Schistosomes and the hygiene hypothesis

While major attempts at de-worming in schistosome endemic areas are important and worthwhile public health goals, there are substantial epidemiological and experimental data that indicate that this could lead to an increased risk for autoimmune diseases and allergy. The “hygiene hypothesis” asserts that the measurable increase in both T_H_1 and T_H_2 immune-mediated diseases that has occurred in the last century in developed nations is a result of improvements in sanitation that have led to increased sensitivity of the immune system to self-antigens and/or allergens (Gale, 2002; Airaghi and Tedeschi, 2004; Rook, 2012). Although the hygiene hypothesis was originally focused on the opposing roles of T_H_1 and T_H_2 immune responses that were established in childhood on later immune challenges, the hypothesis came under scrutiny when it was realized that both T_H_1 (type 1 diabetes) and T_H_2 (allergy) immune-mediated inflammatory diseases were rising in the same geographical areas and demographic groups (Stene and Nafstad, 2001). More telling was the fact that the prevalence of asthma and schistosome infections did not overlap despite both provoking heavily T_H_2-biased immune responses (Yazdanbakhsh et al., 2001). Now that it is understood that chronic helminth infections also stimulate a highly regulatory immune response that affects both T_H_1 and T_H_2 reactions, a role for schistosomes and other worms in the hygiene hypothesis fits better with the epidemiological data (Wynn et al., 1997; Araujo et al., 2004; Maizels, 2009). This intriguing correlation has led to series of experiments in mice as well as clinical trials to test the ability of helminths to modulate autoimmune diseases and asthma.

### Autoimmune diseases and schistosomes

Type 1 diabetes (T1D) is an autoimmune disease that has risen in prevalence in developed and developing nations as hygiene has improved, and is rarely found in areas endemic for schistosomiasis (Zaccone et al., 2008). Cercarial infection of NOD mice that spontaneously develop T1D with *Schistosoma mansoni* prior to T1D disease development led to granuloma formation in the pancreas and a delay in hyperglycemia (Cooke et al., 1999). The same study showed that weekly injection of schistosome eggs led to suppression of anti-insulin IgG antibody production and decreased blood glucose levels in comparison to control NOD mice (Cooke et al., 1999). These effects were later shown to be dependent on the timing of administration and could be recapitulated by treatment with soluble antigens extracted from adult worms as well as with SEA (Zaccone et al., 2003). Splenic T cells from egg-sensitized NOD mice produced similar levels of IFNγ, but much higher amounts of IL-4, IL-5, IL-13, and IL-10 in response to SEA challenge than did cells from control mice (Zaccone et al., 2003). Unlike soluble antigens from adult worms, SEA cooperated with bacterial lipopolysaccharide (LPS) in mediating a shift away from IL-12 production and toward IL-10 production in bone marrow-derived DCs from NOD mice (Zaccone et al., 2003). Adoptive transfer of splenocytes from SEA-treated NOD mice failed to induce T1D in NOD. SCID recipients, an effect attributed to TGFβ-dependent induction of FoxP3^+^/GITR^+^/CD25^+^ T_REG_ cells (Zaccone et al., 2003, 2009). Correlative studies involving schistosome infection have been reported in mouse models of Grave's disease and autoimmune arthritis (Nagayama et al., 2004; Osada et al., 2009; Song et al., 2011).

A series of experiments has been conducted in the EAE model of multiple sclerosis in which susceptible mouse strains are immunized with peptides derived from proteolipid protein or myelin oligodendrocyte glycoprotein emulsified in complete Freund's adjuvant. An initial study demonstrated that prior infection of mice with *Schistosoma mansoni* led to reduced incidence of EAE as measured by paralysis and delayed infiltration of CD3^+^ T cells and F4/80^+^ macrophages into the brain and spinal cord (La Flamme et al., 2003). These effects were attributed to reduced levels of TNFα, IFNγ and IL-12 produced by cells from schistosome-infected mice (La Flamme et al., 2003). Very similar results were obtained using schistosome eggs to pretreat mice prior to EAE disease induction (Sewell et al., 2003). In the latter study, egg injection within the first two days after induction of EAE led to a significant delay in the onset of paralysis but became less effective as the time after disease induction increased (Sewell et al., 2003). The EAE model can be mediated by either T_H_1 or T_H_17 cells, which are both sensitive to immune regulation by IL-4, and it was shown that the effects of schistosome eggs on EAE were dependent on the major signaling transcription factor downstream of IL-4 receptor, STAT6 (Sewell et al., 2003). Protection from EAE has also been demonstrated using either unfractionated SEA or purified LNFPIII derived from schistosome eggs (Zheng et al., 2008; Zhu et al., 2012). Despite the findings that a delay in exposure to schistosomes or their products can prevent therapeutic effects on EAE, the data in humans appears to be more promising. A small cohort of patients that had previously been diagnosed with MS and then developed eosinophilia related to intestinal helminth infection was identified in Argentina (Correale and Farez, 2007). These patients had significantly fewer exacerbations of MS upon 5-year follow up than matched control patients who were uninfected (Correale and Farez, 2007). The peripheral blood of the infected MS patients had a higher frequency of IL-10 and TGFβ producing cells than controls, and significantly reduced numbers of IL-12 and IFNγ producers (Correale and Farez, 2007). Further studies of this cohort indicated that IL-10 producing B cells were prevalent in the infected MS patients (Correale et al., 2008). B cells and DCs from infected patients expressed TLR2 at high levels and suppressed T_H_1 and T_H_17 cell cytokine production while promoting T_H_2 cytokines after treatment with TLR2 ligands (Correale and Farez, 2009).

It has also been suggested that the greatly increased incidence of the inflammatory bowel diseases (IBD), ulcerative colitis and Crohn's disease, in developed countries may be due to reduced exposure to helminths including *Schistosoma mansoni* (Weinstock and Elliott, 2009; Elliott and Weinstock, 2012). The gut mucosa is a site of exposure of the host to many potentially harmful microbes, as well as a majority of non-harmful commensal organisms. In order to maintain a balanced immune response in the gut mucosa, there are specialized lymphoid structures and cell populations that generally have a highly regulatory phenotype (Rubtsov et al., 2008; Maynard and Weaver, 2009). The development of IBD is known to be strongly suppressed by IL-10 and regulatory T cells as evidenced by animal models involving circumvention of these mechanistic pathways (Rubtsov et al., 2008; Glocker et al., 2009). As noted above, both IL-10-producing CD5^+^ B cells and FoxP3^+^ T_REG_ cells are stimulated by infection with schistosomes. In particular, CD5^+^ B cells are normally resident in the mucosa and have high regulatory potential due to inducible expression of IL-10, FasL, regulatory natural antibodies and other mechanisms of immune regulation (Klinker and Lundy, 2012). Direct experimental evidence of a role for schistosome and other helminth infections in the suppression of IBD has come from experimental mouse models (Smith et al., 2007; Weng et al., 2007). Mice that were infected with cercariae 8 weeks prior to the elicitation of colitis in the dextran sodium sulfate (DSS) model did not display weight loss, bloody stools or shortening of colon length as was detected in uninfected control mice (Smith et al., 2007). In addition to induction of IL-10 and TGFβ, neither of which was solely responsible for protection from colitis, schistosome infection led to increased infiltration of macrophages into the intestinal lamina propria that were required for immune suppression (Smith et al., 2007). Although exposure to schistosome eggs did not elicit protection from orally-induced DSS colitis, this treatment was effective in the trinitrobenzene sulfonic acid (TNBS) model that involves the more localized rectal administration of the eliciting agent (Elliott et al., 2003). Similarly, administration of worm proteins extracted from adult schistosomes in the TNBS colitis model as much as 6 h after rectal administration of TNBS led to decreased inflammation (Ruyssers et al., 2009). The worm antigen treatment was accompanied by increased colonic expression of IL-10, TGFβ, and IL-5 but diminished expression of IFNγ, IL-12, IL-17, and IL-4 (Ruyssers et al., 2009). The epidemiological and experimental data outlined above was used as justification for Phase I open-label trials and Phase II randomized, controlled clinical trials using ingestion of ova from the pig whipworm, *Trichuris suis*, to treat ulcerative colitis (Summers et al., 2003, 2005). Oral intake of 2500 *T. suis* ova biweekly for 12 weeks led to clinical improvement in 43% of ova-treated patients compared to 17% improvement using the same measures in placebo-control recipients at the end of the 12 weeks treatment period (Summers et al., 2005). Therapy with *T. suis* eggs may have several advantages over similar treatment with eggs from other helminths since the *T. suis* eggs are easy to obtain and store, and do not permanently colonize humans or invade into tissues where they could become pathogenic (Weinstock et al., 2002).

### Asthma

There was an early expectation that an association of increased incidence of allergic asthma and other atopic disease responses in individuals with schistosoma infections would exist, due to the strongly skewed T_H_2 immune response environment induced by SEA. However, this was not the case and individuals with schistosoma infection were shown to have lower incidence of allergic asthma compared to those without (Yazdanbakhsh et al., 2001; Araujo and De Carvalho, 2006). While this effect does not appear to be limited to schistosomes, they are the most widely endemic infectious organism among helminths and the most thoroughly studied. Initial data suggested that the responses could be correlated to increased levels of IL-10 and subsequently to T_REG_ cells that are generated during chronic schistosoma infection (Cardoso et al., 2006; Yang et al., 2007). Interestingly, recent data immunizing animals to a specific schistosome antigen, Sm22.6, demonstrated that allergen specific IgE as well as T_H_2 cytokines were reduced and correlated with generation of T_REG_ cells (Cardoso et al., 2010). However, subsequent additional studies have demonstrated that numerous B cell associated responses may also contribute to a regulatory environment for the development and regulation of allergic responses, including both B cell-derived IL-10 production and altered T cell activation (Amu et al., 2010; Van Der Vlugt et al., 2012). In these studies, B cell transfer experiments demonstrated that CD1d^hi^ B cells from schistosome-infected animals could modulate allergen-induced responses in an IL-10-dependent manner that stimulated T_REG_ cells in the recipients (Amu et al., 2010; Van Der Vlugt et al., 2012). A similar population of CD1d^hi^ IL-10 producing B cells was found in the peripheral blood of *Schistosoma haematobium*-infected Gabonese children compared to uninfected children (Van Der Vlugt et al., 2012). Importantly, in the latter study a reduction in the B_REG_ population was reduced after treatment of the schistosome infection (Van Der Vlugt et al., 2012). In some B cell transfer studies involving cells isolated from helminth infected mice, there were also notable suppressive effects on asthma that were independent of IL-10, but the mechanisms were not determined (Wilson et al., 2010; Van Der Vlugt et al., 2012). Using animal models of chronic allergic responses it was observed that lung CD5^+^ B cells could express FasL and regulate allergic responses by a FasL-mediated mechanism similar to that previously observed in schistosome infection (Lundy and Boros, 2002; Lundy et al., 2005). What is not clear from these studies is how the regulated immune response controlled by B cells from schistosome infections is able to cross react to allergens and elicit non-specific regulatory functions. Interestingly, since B cells can provide a T_H_2-skewed APC function during allergic responses, there may also be other aspects of B cell biology that alters the direction of T_H_2 type allergic immune responses (Lindell et al., 2008). It is conceivable that one aspect of B cell regulation might be related to the ability to alter the cytokine environment and indirectly affect activation of regulatory T cells. Thus, the infection with Schistosoma has a systemic effect on the development of allergic responses that includes an interrelated generation of numerous regulatory lymphocyte populations, both B and T cells, that may impact several aspects of immunity including vaccination responses.

## Therapeutic potential of schistosome-derived immunomodulators

A challenge moving forward with efforts to eradicate schistosomes will be to find ways of selectively replacing their potentially beneficial immune modulatory effects on allergy and autoimmunity, while preserving immune responses toward other pathogenic microorganisms. The correlations between chronic exposure to schistosome eggs and immune downregulation have led to interest in finding egg-derived molecules that can therapeutically mediate suppression of immune responses (Harnett and Harnett, 2010). It has long been recognized that SEA is a major mediator of the shift in immune response from the proinflammatory and T_H_1 reactions prevalent early after infection toward the T_H_2-dominated response that prevails in the acute phase of egg granuloma formation. SEA is a highly complex mixture of components including proteins, nucleic acids, lipids, glycolipids, complex carbohydrates, and glycoprotein molecules that can have direct or indirect effects on both innate and adaptive immune responses. Schistosome LNFPIII and glycoprotein ω-1 have been particularly interesting candidate molecules that contribute to immune modulation, yet there may be many other molecules in SEA or derived from other helminths that could hold therapeutic potential (Harnett and Harnett, 2010).

### Lacto-N-fucopentaose III

**Figure 2:**
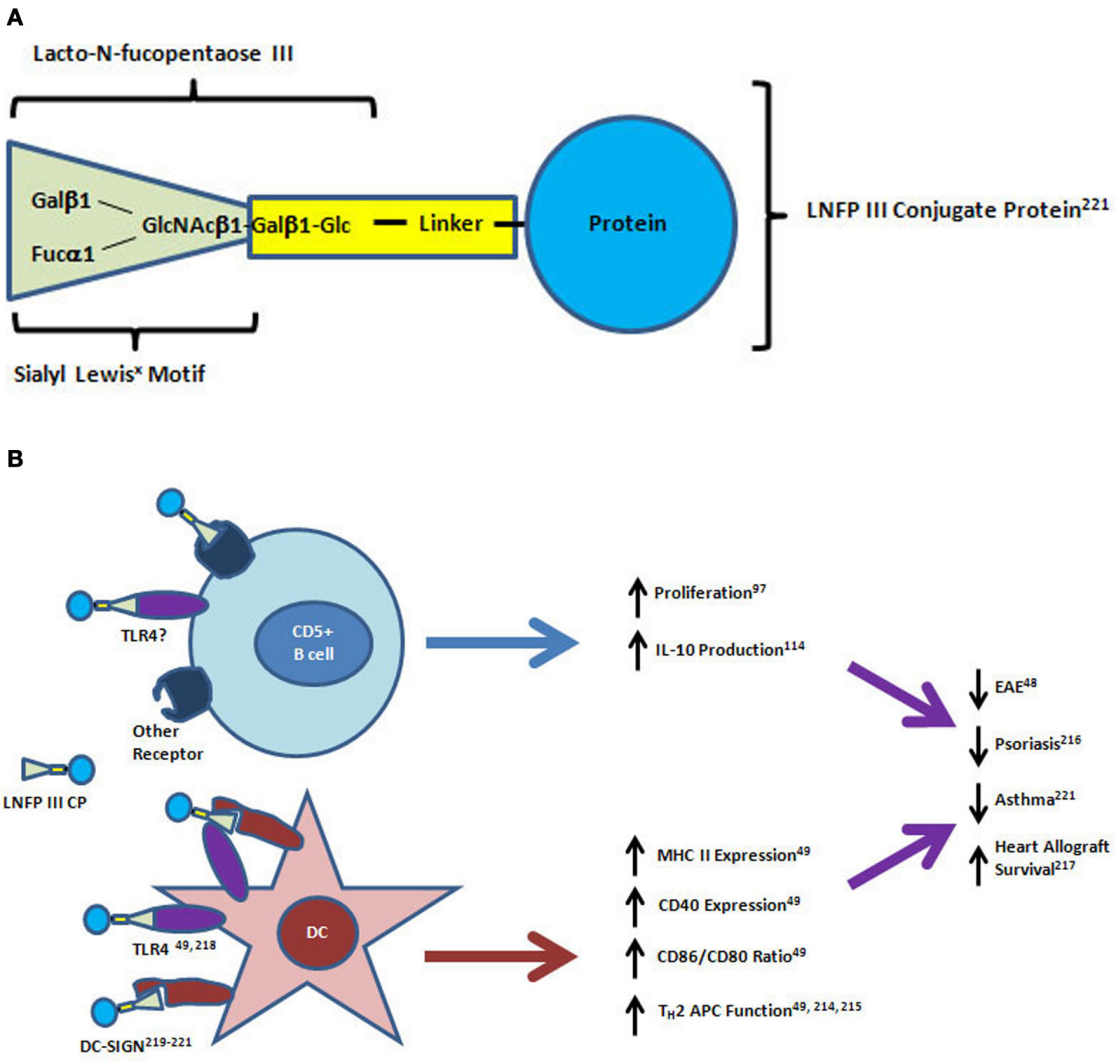
**Structure and mechanisms of action of lacto-N-fucopentaose III protein conjugates on CD5+ B cells and dendritic cells. (A)** The lacto-N-fucopentaose III (LNFPIII) molecule is a pentameric glycan consisting of the trimeric sialyl Lewis^x^ motif (green triangle) linked to a beta-galactose and glucose dimer. For *in vitro* and *in vivo* studies, the glycan conjugated proteins have been purified from soluble egg antigen (SEA) or manufactured by linkage to a carrier protein molecule (bovine serum albumin, dextran, ovalbumin) using biochemical crosslinkers. **(B)** LNFPIII conjugates have direct effects on proliferation and IL-10 production by CD5^+^ B cells. Dendritic cells (DC) respond to LNFPIII with altered antigen presenting capacity that favors T_H_2 induction. Activation of DC by LNFPIII is mediated through TLR4 and/or DC-SIGN, while the receptors for LNFPIII on CD5^+^ B cells have not yet been identified. Treatment of mice with LNFPIII protein conjugates has led to decreased disease progression in experimental autoimmune encephalomyelitis (EAE), asthma and psoriasis models, and led to prolonged heart allograft survival in a transplantation model. DC-SIGN, dendritic cell-specific intracellular adhesion molecule-3-grabbing non-integrin; TLR4, toll-like receptor 4; APC, antigen presenting cell.

The pentameric, sialyl Lewis^x^ containing glycan, lacto-N-fucopentaose III (LNFPIII, CD15), is a major carbohydrate component of SEA (Figure 2A). It was originally identified by antibody binding on human tumor cells and later found to be expressed by many human adult and fetal tissues, particularly in the mucosa (Brockhaus et al., 1982; Combs et al., 1984; Velupillai and Harn, 1994). As described above and shown in Figure 2B, the LNFPIII found in SEA was originally shown to have direct effects on the stimulation of IL-10 production by CD5^+^ B cells *in vitro* (Velupillai et al., 1997). LNFPIII also drives a strong T_H_2 response toward protein antigens with which it is conjugated when injected into mice (Okano et al., 2001; Faveeuw et al., 2002). Subcutaneous injection of a dextran-conjugated LNFPIII compound into psoriasis susceptible fsn/fsn mice prior to disease onset was shown to have a preventive effect on the formation of skin lesions (Atochina and Harn, 2006). LNFPIII has also been shown to be therapeutic in a mouse model of multiple sclerosis, and it prolonged the survival of allogeneic heart transplants in mice (Dutta et al., 2010; Zhu et al., 2012). LNFPIII skews the antigen presenting capacity of dendritic cells (DC) toward CD4^+^ but not CD8^+^ T cells (Wang et al., 2010). Treatment of spleen-derived DC from naïve mice with LNFPIII led to increases in MHC Class II and CD40 surface expression similar to the effects seen after bacterial lipopolysaccharide (LPS) treatment (Wang et al., 2010). However, LNFPIII induced higher expression of CD86 and lower expression of CD80 than LPS resulting in a dramatic difference in the ratio of these agonists for CD28 and CTLA4 molecules expressed by T cells (Wang et al., 2010). In coculture experiments, the LNFPIII treated DC preferentially induced IL-4 production from OT-II transgenic T_H_ cells isolated from the spleens of naïve mice (Wang et al., 2010). Details of the signaling effects downstream of LNFPIII treatment in DC has recently been well reviewed (Harnett and Harnett, 2010). LNFPIII signaling is dependent on TLR4, the receptor for LPS, but downstream signaling is significantly different between the two ligands particularly in the ERK and MAP kinase pathways and the mechanism of activation of the transcription factor NFκB (Thomas et al., 2005; Wang et al., 2010). In contrast to LPS stimulation, LNFPIII signals independently of MyD88 and does not induce degradation of the inhibitor of NFκB (Thomas et al., 2005). A putative mechanism for this difference involves the binding of LNFPIII to C-type lectin receptors such as DC-specific ICAM-3 grabbing non-integrin (DC-SIGN) by the Lewis^x^ motif (Van Liempt et al., 2004, 2007; Singh et al., 2009). It has been shown that activation of C-type lectins through binding of antibodies that are glycosylated with Lewis^x^ containing glycans is a mechanism of immune suppression induced by IVIG treatment, a therapy for immune thrombocytopenic purpura and other diseases (Anthony et al., 2008, 2012).

### Glycoprotein ω-1

In the case of treatment for autoimmune diseases that are mediated by T_H_1 or T_H_17 immune responses, it may be sufficient to skew the response toward the T_H_2 cytokine, IL-4, which has an inhibitory effect on IL-12 and IL-17 production. The SEA-derived T2 ribonuclease omega-1 (ω-1) has a direct effect on antigen presenting cells that supports T_H_2 polarization (Everts et al., 2009, 2012; Steinfelder et al., 2009). Purified and recombinant ω-1 suppressed the ability of LPS to induce IL-12 secretion from DC as well as their ability to induce IFNγ-producing T_H_1 cells in coculture experiments (Everts et al., 2009; Steinfelder et al., 2009). Injection of purified ω-1 into 4get/KN2 mice demonstrated that ω-1 could prime T_H_2 responses *in vivo* and further experiments showed that T_H_2 induction was independent of the IL-4R suggesting that the effects of ω-1 on T_H_2 priming were direct (Everts et al., 2009). Like LNFPIII, ω-1 is glycosylated with sialyl Lewis^x^ motifs, and the T_H_2 inducing capacity of ω-1 was shown to be dependent on glycosylation as well as the ribonuclease activity of the protein (Meevissen et al., 2010; Everts et al., 2012). Treatment of DC with SEA or purified ω-1 glycoprotein caused changes to the adherence of DC to plastic dishes and disrupted interactions with antigen-specific T_H_ cells (Steinfelder et al., 2009). Antibody neutralization and studies performed in mice with deficiencies in the mannose receptor indicated that ω-1 was dependent on the mannose receptor, but not on DC-SIGN, for uptake into DC *in vitro* and T_H_2 polarization *in vivo* (Everts et al., 2012). Treatment of NOD mice with ω-1 led to global increases in the proportion of T_H_1, T_H_17, and T_H_2 cells compared to untreated control mice, as well as a marked induction of FoxP3^+^CTLA4^+^ T_REG_ cells (Zaccone et al., 2011). The induction of FoxP3 expression by ω-1 was dependent on the functions of TGFβ and retinoic acid (Zaccone et al., 2011). It has not been reported whether treatment with ω-1 causes reduced or delayed development of diabetes in the NOD mice. Thus, both the glycoprotein ω-1 and the glycan LNFPIII components purified from SEA have demonstrable effects on adaptive immune parameters making each molecule a strong candidate for development of helminth-based immune therapeutics.

## Summary and concluding remarks

Although nearly every person may have been expected to be infected by a helminth in their lifetime just a century ago, heroic eradication efforts have all but eliminated helminth infection from many areas and brought the infection rate down to an estimated 10–20% of people worldwide. This has led to vast improvements in the quality of life for billions of people, and will have great benefits for combatting other major infections. Several regulatory populations of lymphocytes are activated as the infection becomes chronic and these diminish the responses of schistosome antigen-specific effector T helper cells, and limit granuloma formation around newly deposited eggs. The chronic nature of schistosome-induced immune modulation can also affect immune responses toward other antigen sources, leading to poorer reactions to subsequent infections by other microbes. Also accompanying the shift in worm infection rates have been measurable increases in the rates of hyper-inflammatory diseases including allergies, asthma, and many autoimmune diseases. Chronic helminth infections may drive immune suppression toward allergens and self-antigens, thus potentially explaining the lower incidence of allergies, asthma and autoimmunity in schistosome endemic areas. Studies of the cellular and molecular mechanisms underlying schistosome-induced immune suppression in mice have supported the importance of regulatory T lymphocytes and the effector cytokine TGFβ as mediators of suppression. The schistosome infection model has also been instrumental in the discovery of immune regulatory functions of B cells including IL-10 production, and expression of the death-inducing molecule Fas ligand. IL-10-producing B cells may mediate cooperative effects with regulatory T cells, while FasL^+^ B cells have been shown to have antigen-specific killer effects on T helper cells in models of asthma and autoimmunity. The data from adoptive cell transfer studies gives striking evidence that schistosomes do have cross-regulatory effects on autoimmunity and asthma. The goal as we move forward will be to find ways to mimic or replace the protective aspects of helminth infections, while continuing to improve hygienic conditions in endemic areas.

### Conflict of interest statement

The authors declare that the research was conducted in the absence of any commercial or financial relationships that could be construed as a potential conflict of interest.
